# Overexpression of △12, △15-Desaturases for Enhanced Lipids Synthesis in *Yarrowia lipolytica*

**DOI:** 10.3389/fmicb.2020.00289

**Published:** 2020-02-25

**Authors:** Feng Xin Yan, Gui Ru Dong, Shan Qiang, Yong Jie Niu, Ching Yuan Hu, Yong Hong Meng

**Affiliations:** ^1^College of Mechanical and Electronic Engineering, Northwest A&F University, Yangling, China; ^2^Shaanxi Engineering Laboratory for Food Green Processing and Security Control, College of Food Engineering and Nutritional Science, Shaanxi Normal University, Xi’an, China; ^3^Xi’an Healthful Biotechnology Co., Ltd., Xi’an, China; ^4^Department of Human Nutrition, Food and Animal Sciences, College of Tropical Agriculture and Human Resources, University of Hawaii at Manoa, Honolulu, HI, United States

**Keywords:** lipid production, metabolic engineering, *Yarrowia lipolytica*, fatty acid desaturases, unsaturated fatty acids

## Abstract

Microbial oil triacylglycerol (TAG) from the renewable feedstock attract much attention. The oleaginous yeast *Yarrowia lipolytica* has become the most studied for lipid biosynthesis. Fatty acid desaturases catalyze the introduction of a double bond into fatty-acid hydrocarbon chains to produce unsaturated fatty acids. Desaturases are known to enhance lipid accumulation. In this study, we have achieved a significant increase in lipid production and increase the unsaturated fatty acids content in *Y. lipolytica*. By comparing the expression of the native genes of △-9 stearoyl-CoA desaturase (SCD) and △12 desaturase (△12D), and an exogenous △15 desaturase (△15D) from flax in the strain with deleted peroxisomal biogenesis factor 10 (PEX10) and overexpressed diacylglyceride acyl-transferase (DGA1), we found that the strain with overexpressed △15 desaturase accumulated 30.7% lipid. Simultaneously, we explored the effect of two copies of desaturase genes (*12D*-*SCD*, *15D*-*SCD*, *12D*-*15D*) on lipid production, and found co-expression of △*12D* and △*15D* accumulated 42.6% lipid. The lipid content was further increased by 56.3% through the deletion of the multifunctional enzyme (MFE1) and the overexpression of acetyl-CoA carboxylase (ACC1). Finally, the lipid productivity of 50 g/L and maximal lipid content of 77.8% DCW are obtained using a 5-L stirred-tank bioreactor during the stationary phase in the engineered YL-10. Our result demonstrated that the △12 and △15 desaturases play an important role in lipid production in *Y. lipolytica* and provides an effective strategy for biodiesel development.

## Introduction

Biofuels are a promising alternative to reduce the dependence on fossil fuels and alleviate environmental pressure (Stephanopoulos, 2007). The bio-based system provides a particularly attractive platform for the sustainable production of biodiesel. In particular, microbial oil triacylglycerol (TAG) from the renewable feedstock attracted much attention because TAG can be directed toward fatty acid ester and alkane production (Blazeck et al., 2013; Beopoulos et al., 2014; Wang et al., 2016). Among oleaginous microorganisms, *Yarrowia lipolytica* is the most studied due to both its unusual biotechnological characteristics and its suitability to genetic manipulation in the laboratory (Ledesma-Amaro and Nicaud, 2015). Using the oleaginous yeast *Y. lipolytica* in a semi-continuous system and using dilute acetic acid as the sole carbon source, superior lipid titer (115 g/L) and productivity (0.8 g/L/h) have been achieved, which demonstrates that *Y. lipolytica* has great potential for biodiesel production (Xu et al., 2017).

Metabolic engineering of *Y. lipolytica* provides a unique platform for lipids and biofuel production. Many strategies were used for increasing intracellular lipid accumulation. First, the deletion of either six acyl-CoA oxidase enzymes (POX1-6), the multifunctional enzyme (MFE1), and the thiolase POT1 (YALI0E18568g) involved in peroxisomal β-oxidation or peroxisomal biogenesis factor 10 (PEX10) resulted in blocking the fatty acid degradation pathway, consequently, increased lipid accumulation (Dulermo and Nicaud, 2011; Tai and Stephanopoulos, 2013; Ledesma-Amaro and Nicaud, 2015). Second, increasing lipid biosynthesis can be achieved through enhancing the supply of pathway precursors, which is primarily initiated by the activity of the malic enzyme (ME), ATP citrate lyase (ACL), and acetyl-CoA carboxylase (ACC1) (Tai and Stephanopoulos, 2013; Zhang et al., 2013; Blazeck et al., 2014; Ledesma-Amaro and Nicaud, 2015). Third, elevated expression of the key enzymes DGA1 and DGA2 (acyl-CoA: diacylglycerol acyl-transferases I and II) are known to promote TAG biosynthesis and transport TAG into the lipid droplet (Gajdoš et al., 2015; Qiao et al., 2015; Friedlander et al., 2016).

Besides the above strategies, a novel fatty acid metabolic regulator, stearoyl-CoA desaturase (SCD and △9D), through the reverse engineering of the mammalian fat-storing pathway, has been identified. Overexpression of this particular desaturase led to the enrichment of intracellular monounsaturated fatty acids and lipid accumulation (Qiao et al., 2015). Fatty acid desaturases, which are membrane-bound proteins, catalyze the introduction of a double bond into fatty-acid hydrocarbon chains to produce unsaturated and polyunsaturated fatty acids (PUFA) (Bai et al., 2016; Díaz et al., 2018). The fatty acid synthetic pathway includes a variety of desaturases, which are specific to the location, number, and stereochemistry of double bonds present in the fatty acids (Fickers et al., 2003). For example, SCD (△9D) is an endoplasmic reticulum-bound enzyme that catalyzes the first step in the PUFA biosynthetic pathway. This SCD introduces a double bond at the carbon 9 of stearic acid (C18:0) to generate oleic acid (C18:1). △12 desaturase introduces a double bond between the 12 and 13 carbon atom of the fatty acid chains. It primarily regulates the synthesis and content of oleic acid (OA), and linoleic acid (LA, 18:2) (Ledesma-Amaro and Nicaud, 2015). The expression of a bifunctional △12/ω3 desaturase from the *Fusarium moniliforme* in both *Y. lipolytica* and soybean resulted in increased ω3 desaturation and the α-linolenic acid (ALA, 18:3 ω3)/LA ratio, and significantly improved the production of ω3 long-chain polyunsaturated fatty acids (LC-PUFAs) (Damude et al., 2006). Co-expression of △6- and △12-desaturase genes allowed for significant production of γ-linolenic acid (GLA) (Chuang et al., 2010). Additionally, expression of △15 desaturase gene (AEP37840) from flax in *Lipomyces starkeyi* showed the successful conversion of LA into ALA and fatty acid accumulation (Salunke et al., 2015).

Overexpression of SCD in *Y. lipolytica* by introducing a native copy of the *SCD* gene, in the strain overexpressing *DGA1* and *ACC1*, showed a significant growth advantage and yielded a higher lipid titer of 55 g/L in *Y. lipolytica* (Qiao et al., 2015). However, this report identified only the SCD as the fatty acid regulator from specialized mammalian cell types, which has a similar oleaginous phenotype in yeast (Qiao et al., 2015). We would like to know whether other desaturases in the *Y. lipolytica* has a similar role as the SCD, and whether increasing the copy number of the SCD and other desaturases enhance the lipid accumulation and increase the unsaturated fatty acids content.

In this study, we compared the expression of the native genes of *SCD* (YALI0C05951g), △*12D* (YALI0B10153g), and an exogenous △*15D* from flax in a strain with deleted *PEX10* (YALI0C01023g) and overexpressed *DGA1* (YALI0E32769). We found that all these desaturases increased lipid production. △15 desaturase, in particular, showed a notable increase in lipid content. Simultaneously, we explored the effect of two copies of each desaturase genes (*12D*-*SCD*, *15D*-*SCD*, and *12D*-*15D*) on lipid production. The results showed that two copies of △12 and △15 desaturases accumulated more lipid synthesis. The combination of *MFE1* (YALI0E15378g) deletion and *ACC1* (YALI0C11407g) overexpression in *Y. lipolytica* led to a superior lipid producer. This study demonstrated that the △12 and △15 desaturases in *Y. lipolytica* play an important role in lipid production, and through the manipulation of these two desaturases, it provides an effective strategy for biodiesel development.

## Materials and Methods

### Strains and Media

*E*scherichia *coli* DH5α was used for cloning and plasmid propagation. DH5α was grown at 37°C with constant shaking in Luria–Bertani Broth (LB) supplemented appropriate antibiotics (ampicillin 100 μg/mL). The *Y. lipolytica* strain PO1f (ATCC # MYA-2613), a leucine and uracil auxotroph devoid of any secreted protease activity (Madzak et al., 2000), was used as the strain for all studies. All strains, plasmids, genes, and enzymes used in this study are listed in Table 1 and Supplementary Table 1.

**TABLE 1:** Strains and plasmids used in this study.

**Strains**	**Characteristics**	**Source**
*E*scherichia *coli* DH5α	*endA1 hsdR17* [r-m + ] *supE44 thi-1 recA1 gyrA* [NalR] *relA relA1* Δ[*lacZYA-argF*] *U169 deoR* [Ø80Δ (*LacZ*) M15]	Novagen
PO1f	MatA, leucine^–^, uracil^–^, xpr2-322, axp1-2	Madzak et al., 2000
YL-1	MatA, leucine^+^, uracil^–^, xpr2-322, axp1-2, △*PEX10*	This work
YL-2	YL-1, △*PEX10*, *DGA1*	This work
YL-3(SCD^+^)	YL-1, *DGA1*, *SCD*	This work
YL-4(12D^+^)	YL-1, *DGA1*, *12D*	This work
YL-5(15D^+^)	YL-1, *DGA1*, *15D*	This work
YL-6(12D^+^-SCD^+^)	YL-1, *DGA1*, *12D*, *SCD*	This work
YL-7(15D^+^-SCD^+^)	YL-1, *DGA1*, *15D*, *SCD*	This work
YL-8(12D^+^-15D^+^)	YL-1, *DGA1*, *15D*, *12D*	This work
YL-9	YL-1, △*MFE1*:*12D*-*15D*-*DGA1*	This work
YL-10	YL-9, *ACC1*	This work
**Plasmids**	**Relative characteristics**	**Source**
pJN44	Expression vector, leu^+^, cEN 1-1, TEF, Introl, xpr2 terminator	Wang et al., 2016
pJN43	TEF, Introl, xpr2 terminator	Wang et al., 2016
pJN43-SCD	P_TEF_-*SCD*	This work
pJN43-*12D*	P_TEF_-*12D*	This work
pJN43-*15D*	P_TEF_-*15D*	This work
pJN44-*DGA1*	P_TEF_-*DGA1*	This work
pJN44-*ACC1*	P_TEF_-*ACC1*	This work
pJN44-*DGA1*-*SCD*	P_TEF_-*DGA1*, P_TEF_-*SCD*	This work
pJN44-*DGA1*-*12D*	P_TEF_-*DGA1*, P_TEF_-*12D*	This work
pJN44-*DGA1*-*15D*	P_TEF_-*DGA1*, P_TEF_-*15D*	This work
pJN44-*DGA1*-*12D*-*SCD*	P_TEF_-*DGA1*, P_TEF_-*12D*, P_TEF_-*SCD*	This work
pJN44-*DGA1*-*15D*-*SCD*	P_TEF_-*DGA1*, P_TEF_-*15D*, P_TEF_-*SCD*	This work
pJN44-*DGA1*-*12D*-*15D*	P_TEF_-*DGA1*, P_TEF_-*12D*, P_TEF_-*15D*	This work
pLoxp-ura-loxp	Knock out vector, loxp, ura3, ura3-testR1, ura3-F, loxp, AmpR	Wang et al., 2016
p*PEX10*-up-ura3-loxp- down	*PEX10*-up, Loxp, ura3, ura3-testR1, ura3-F, loxp, *PEX10*-down, AmpR	This work
p*MFE1*-up-ura3-loxp- down	*MFE1*-up, Loxp, ura3, ura3-testR1, ura3-F, loxp, *MFE1*-down, AmpR	This work
ura-△*MFE1*:*DGA1*-*12D*-*15D*	△*MFE1*:*12D*-*15D*-*DGA1* in vector p*MFE1*-up-ura3- loxp-down, AmpR	This work

The engineered *Y. lipolytica* strains were selected using synthetic complete (SC) medium, which consisted of 1.7 g/L Yeast Nitrogen Base (YNB) (without amino acids and ammonium sulfate), 20 g/L glucose, 5 g/L (NH_4_)_2_SO_4_, and supplemented with appropriate amino acid dropout mix. SD-URA contained 2 g/L drop-out mix synthetic minus uracil, and SD-LEU contained 2 g/L drop-out mix synthetic minus leucine (United States Biological; Marblehead, United States). Lipid accumulation media contained 20 mg/L uracil (when the strain was uracil auxotroph), 80 g/L glucose, 1.7 g/L Yeast Nitrogen Base, and 0.352 g/L (NH_4_)_2_SO_4_.

### Construction of Plasmids

Plasmids pJN43 (P_TEF_-Txpr2) and pJN44 (P_TEF_-Txpr2) were used for gene episomal expression in the study in *Y. lipolytica* (Wang et al., 2016). They are centromeric and replicative vector. Plasmid pJN44 contains the leucine selection marker, whereas pJN43 does not. The maps for pJN44 and pJN43 used in this study are listed in Supplementary Figure 1. The following genes were PCR-amplified from *Y. lipolytica* total DNA as the template with primers listed in Supplementary Table 2. The PCR products △-9 stearoyl-CoA desaturase gene (*SCD*), △12 desaturase gene (△*12D*), or synthesized fragment [△15 gene (△*15D*, AEP37840) from flax codon-optimized] were inserted into vectors of pJN43 (pJN44 was digested with *Xba*I and then self-ligated to remove the leucine marker) with a *Hin*dIII/*Sma*I digest except *SCD* (*Hin*dIII/*Pst*I), resulting in pJN43-*SCD*, pJN43-*12D*, and pJN43-*15D*. Diacylglyceride acyl-transferase gene (*DGA1*) and acetyl-CoA carboxylase gene (*ACC1*) were PCR-amplified from *Y. lipolytica* DNA and inserted into vector pJN44 with a *Hin*dIII/*Sma*I digest (*DGA1*) or Gibson method using Seamless Cloning and Assembly Kit (TransGen Biotech; Beijing, China). This formed plasmids pJN44-*DGA1*, pJN44-*ACC1*.

For the construction of plasmid pJN44-*DGA1*-*12D*, the fragment of *12D* was obtained from pJN43-*12D* with an *Xba*I/*Spe*I digest and was inserted into a pJN44-*DGA1* plasmid with a *Spe*I/Fast Alkaline Phosphatase (FastAP) digest (Wang et al., 2016). For the construction of plasmid pJN44-*DGA1*-*12D*-*15D*, the fragment of *15D* was obtained from pJN43-*15D* with an *Xba*I/*Spe*I digest and was inserted into a pJN44-*DGA1*-*12D* plasmid with a *Spe*I/Fast Alkaline Phosphatase (FastAP) digest. The construction of the remaining plasmid expression cassette was performed similarly.

Plasmids pLoxp-ura-loxp (the map could be found in Supplementary Figure 2) for gene knockout contained the uracil selection marker surrounded by LoxP sites (Wang et al., 2016). To construct plasmid p*MFE1*-up-ura3-loxp-down or p*PEX10*-up-ura3-loxp-down, the 5′ and 3′ flanking regions of corresponding genes were amplified from *Y. lipolytica* DNA with the primers listed in Supplementary Table 2, and the amplicon was inserted into the upstream and downstream of a uracil selection marker in plasmid pLoxp-ura-loxp, respectively (Wang et al., 2016). To construct integrated expression cassette plasmid ura-△*MFE1*:*DGA1*-*12D*-*15D*, the fragment of *DGA1*-*12D*-*15D* was obtained from pJN44-*DGA1*-*12D*-*15D* with an *Xba*I/*Spe*I digest and was inserted into a p*MFE1*-up-ura3-loxp-down plasmid with a *Spe*I/FastAP digest.

### Strain Construction

Knockout mutants were constructed by homologous recombination (transformation with linearized knockout cassette) and marker rescue (Cre-recombinase based uracil marker deletion) as previously described (Fickers et al., 2003; Wang et al., 2016). We first constructed the strain YL-1 (PO1f-△*PEX10*) through the deletion of *PEX10* in the *Y. lipolytica* PO1f. The strain YL-1was used for episomal expression of pJN44-*DGA1*, pJN44- *DGA1*-*12D*, pJN44-*DGA1*-*SCD*, pJN44-*DGA1*-*15D*, pJN44-*DGA1*-*12D*-*SCD*, pJN44-*DGA1*-*15D*-*SCD*, and pJN44-*DGA1*-*12D*-*15D*, utilizing a selective marker leucine. Then, the integrative cassette plasmid ura-△*MFE1*:*DGA1*-*12D*-*15D* was linearized (*12D*-*15D*-*DGA1*-*MFE1*-up-loxp-down), inserted into the YL-1 strain, and the transformants were screened using uracil dropout plate to create the strain YL-9. Then the plasmid pJN44-*ACC1* was expressed in YL-9, resulting in YL-10. The transformation of *Y. lipolytica* was performed using Zymogen Frozen EZ yeast transformation kit II (Zymo Research Corporation; United States) according to the manufacturer’s instruction.

### Total RNA Isolation and Quantitative PCR Analysis

*Yarrowia lipolytica* and the transformed clones growing for 72 h were collected for RNA isolation using the method as described previously (Tai and Stephanopoulos, 2013). RNA samples were reverse transcribed using EasyScript One-Step gDNA Removal and cDNA Synthesis SuperMix kit (Transgen; Beijing, China) (Qiao et al., 2015). qRT–PCR analyses were carried out using TransStrat Tip Green qPCR SuperMix Kit (Transgen; Beijing, China) and PIKOREAL 96 Real-Time PCR System. Fluorescence results were analyzed using Real-time PCR Miner and relative quantification using *ACTIN* (YALI0D08272g) as the reference gene and PO1f as the reference strain (Tai and Stephanopoulos, 2013). All samples were analyzed in triplicates. The primers are listed in Supplementary Table 2. The relative expression of the gene was analyzed using the 2^–△△ct^ method (Livak and Schmittgen, 2000).

### Lipid Extraction and Quantification

Wet cells were directly methylated to determine fatty acid content and composition according to the following procedure. 1 mL of lipid sample was transferred into 16 mm × 125 mm screw-cap pyrex culture tubes. 1 mL 0.1 mg/mL of C13:0 (Tridecanoic acid) was added as the internal standard. The mixture was incubated at 85°C for 15 min, followed by the addition of 5.3 mL methanol and 0.7 mL 10 M NaOH solutions. Then the solution was neutralized with 0.58 mL H_2_SO_4_; the resulting solution was vortexed and incubated at 85°C for 15 min. 2 mL H_2_O and 2 mL hexane were added, and the mixture was vortexed at room temperature for 15 min to extract the fatty acid methyl esters (FAMEs) for Gas Chromatography-Mass Spectrometer (GC-MS) analysis. After extraction, the mixture is centrifuged at 8000 × *g* for 1 min, and the top hydrophobic phase was transferred to a vial for GC-MS analysis.

Samples were analyzed in a GC-MS (QP2010 Ultra) with a capillary column RTX-5MS (30 m × 0.25 mm × 0.25 mm). The following settings were used: 120°C (1 min), increased at 7°C/min to 250°C, increased at 8°C/min to 295°C, hold at 295°C for 7 min. Helium was used as carrier gas. Mass spectrometer operating conditions were as follows: electron impact energy 70 eV; emission current 250 μA, transfer line 310°C; source temperature 230°C; scan rate 0.8 scans/-s and mass range 40–650 Da. Fatty acids were identified and quantified by comparison with commercial FAME standards normalized to methyl tridecanoate (C13:0). Total lipid content was calculated as the sum of total fatty acid contents for five FAMEs: methyl palmitate (C16:0), methyl palmitoleate (C16:1), methyl stearate (C18:0), methyl oleate (C18:1), and methyl linoleate (C18:2). The addition of tridecanoic acid was used as the internal standard, which was carried through the entire analysis procedure and transesterified into its methyl ester.

### Bioreactor Fermentations

Bioreactor fermentation was carried out in a 5-l baffled stirred-tank bioreactor. The medium used contained 1.7 g/L yeast nitrogen base (without amino acids and ammonium sulfate), 3.52 g/L ammonium sulfate, 2 g/L uracil, and 40 g/L glucose. Oxygen was supplied in the form of filtered air via sparging rate of 1–5 L/min of air using agitation in 200–800 rpm range to maintain dissolved oxygen levels above 20% during growth phase (0–48 h) and 0–5% at stationary phase (Qiao et al., 2015). The temperature was maintained at 28°C, and the pH of the culture was continuously controlled at 5.50 using 10% KOH.

### Confocal Microscopy

Confocal microscopy was used to capture fluorescence images. The engineered strain cells were harvested, washed twice, and suspended in 500 μL Phosphate-Buffered Saline solutions (PBS). The cells in PBS buffer were mixed with the Nile red dye at the concentration of 0.5 mg/mL in dimethyl sulfoxide (DMSO). The final concentration of the Nile red for cell staining was 1 μg/mL. After incubation of the cells with the Nile red for 20 min in the dark at room temperature, the samples were washed and then observed using confocal microscopy. The fluorescence of the Nile red was excited at a wavelength of the 488 nm and detected in the range of 525 nm with a 100 × oil-immersion objective (Beopoulos et al., 2008; Blazeck et al., 2014; Qiao et al., 2015).

### Statistical Analysis

Statistical analyses were performed using SPSS 18.0 (Chicago, IL). All experiments were repeated three times. Data in Figures 2–6 are shown as Mean ± Standard Deviation. Data in Figures 2–5 were analyzed using one-way analysis of variance (ANOVA), and the least significant difference (LSD) was used to separate the means.

## Results

### Combination of the Best Lipid Accumulation Genetic Modifications

**FIGURE 1:**
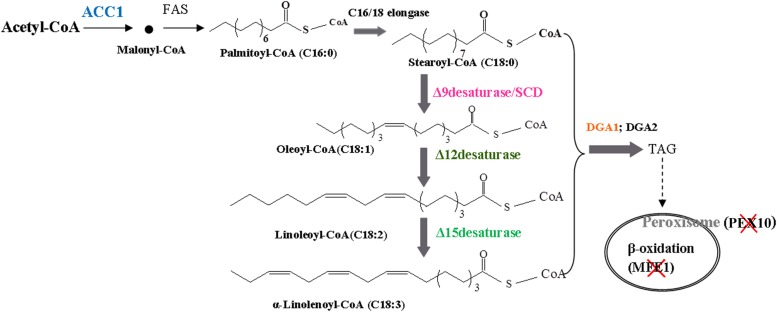
A schematic illustrating the pathways rewired in *Y. lipolytica’s* metabolism to drastically increase lipogenesis capacity. Acetyl-CoA carboxylase gene (*ACC1*): *ACC1* catalyzes the first committed step toward lipid biosynthesis, converting cytosolic acetyl-CoA into malonyl-CoA, which is the primary precursor for fatty acid synthesis. Diacylglycerol acyltransferases I gene (*DGA1*): *DGA* promotes triacylglycerol (TAG) biosynthesis and transport TAG into the lipid droplet. The multifunctional enzyme gene (*MFE1*): *MFE1* catalyzes both the second and third steps of fatty acid β-oxidation. Peroxisome biogenesis factor 10 (*PEX10*), a transcription factor necessary for correct peroxisomal biogenesis and morphology. *SCD*, *12D*, and *15D*: The fatty acid desaturases catalyze the introduction of double bonds into △9, △12, and △15 fatty-acid hydrocarbon chains to produce unsaturated fatty acids.

**FIGURE 2:**
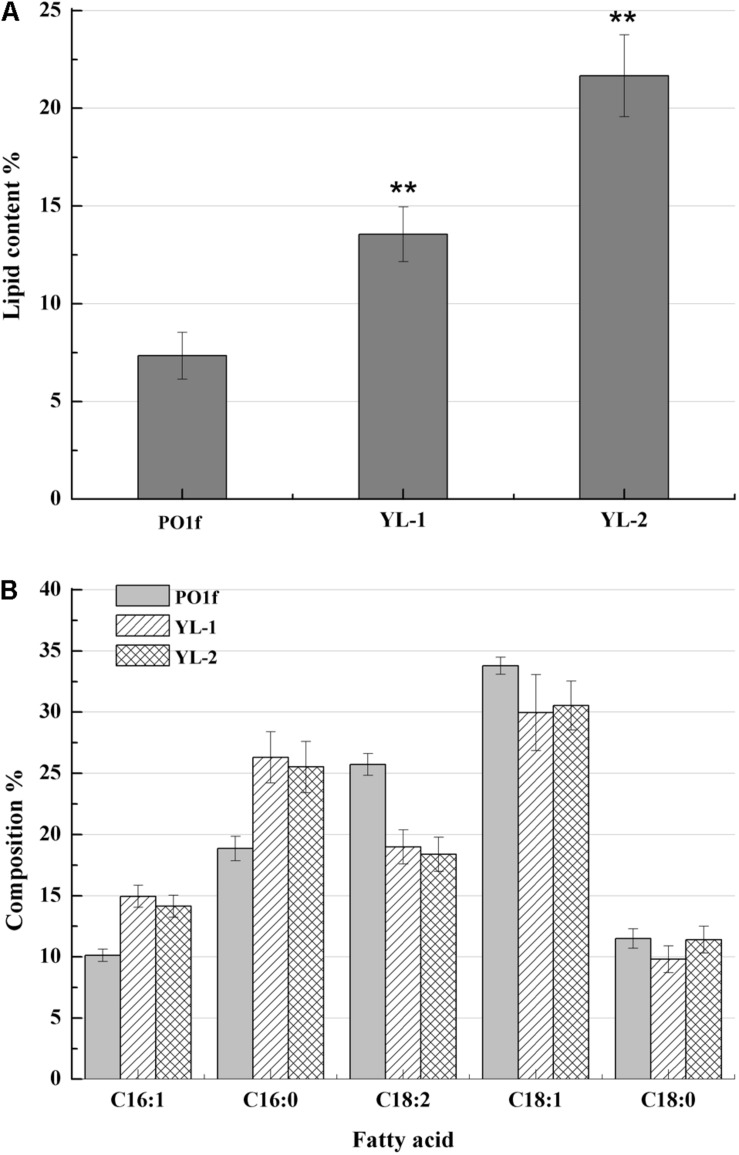
Lipid contents and fatty acid profiles of the strains with *PEX10* deletion and *DGA1* overexpression. **(A)** The percentage of lipid and fatty acid dry cell weight (Lipid% DCW) was shown for strains PO1f, YL-1, YL-2 cultivated in the media of C:N = 50. **(B)** Fatty acid profiles of lipid extract from the strains PO1f, YL-1, YL-2 were shown after 6 day shake flask fermentation. Error bars represent standard deviations (*n* = 3). The asterisks indicate a significant difference compared with the control (**p < 0.01).

The fatty acid biosynthesis pathway and engineered strategies used in this study are depicted in Figure 1. To increase fatty-acid accumulation in *Y. lipolytica*, we used a well-established method used to remove β-oxidation capacity and to overexpress the native *DGA1* (YALI0E32769g). The leucine biosynthetic capacity has been implicated as an effector of lipogenic ability in the oleaginous organisms (Blazeck et al., 2014). Therefore, an empty plasmid pJN44 (a leucine marker) needs to be inserted into the leucine auxotroph strains before the lipid content can be measured. Here, we created the *PEX10* deletion strain (*PEX10* disruption cassette was constructed as described in the Materials and Methods)-, which accumulated lipid at 14.5% of DCW, about 1-fold increase compared to 7.2% for the control strain POlf (Figure 2A). The *PEX10* deletion strain is designated as YL-1.

*DGA1* is the enzyme catalyzes the final step of the triglyceride (TAG) synthesis pathway. *DGA1* is essential for lipid accumulation in *Y. lipolytica*. Therefore, plasmid pJN44-*DGA1* was inserted into YL-1 to generate strain YL-2, which overexpresses *DGA1*. We analyzed the lipid profiles and content of YL-2 with GC-MS and saw C16:1, C16:0, C18:2, C18:1, and C18:0 fatty acids (Figure 2B) predominantly. The strain YL-2 accumulated 22.56% lipid content, a two-fold improvement over control (Figure 2A). This result suggests that there was an additive effect of *PEX10* mutation and overexpression of *DGA1* in lipid accumulation. The fatty acid profiles of PO1f, YL-1, and YL-2 strains were analyzed and presented in Figure 2B. For the strain PO1f, the major fatty acid in the lipid fraction was linoleic acid (C18:2, 23.4%) and oleic acid (18:1, 32.9%). Several other FAs were present at levels of about 10 to 20%. For the strain YL-1, the levels of C18:1 and C18:2 decreased, while levels of C16:0 and C16:1 were increased (Figure 2B). Moreover, the ratio of C16 to C18 acyl chain fatty acid was changed from 0.46 to 0.71, and the ratio of unsaturated to saturated fatty acid decreased from 2.12 to 1.74. *PEX10* has been shown to regulate the composition of intracellular fatty acid content, and deletion of *PEX10* was associated with a decreased linoleic acid content, despite an overall increase in lipid content (Hong et al., 2009). The composition of the total cellular fatty acid of YL-2 was very similar to that of the YL-1 strain.

Based on the results of YL-1 and YL-2, it was clear that we have successfully increased intracellular lipid content utilizing two distinct and additive methods. We also found that the *PEX10* deletion changed the fatty acid composition; especially, the unsaturated fatty acid content was decreased.

### Comparison of the Different Desaturase Genes for Increased Lipid Production

Stearoyl-CoA desaturase (SCD), through the reverse engineering of the mammalian fat-storing pathway, was identified as a metabolic regulator (Qiao et al., 2015). The engineered strain exhibited lipid overproduction and fast cell growth when *SCD* was expressed in the *DGA1* and *ACC1* overexpressing strain of *Y. lipolytica*. Except for *SCD*, the fatty acid synthesis pathway comprises other desaturases in yeast. We know that mammals, including humans, do not contain certain desaturases (△*12D* and △*15D*) for producing essential fatty acids (Lee et al., 2016). However, △*12D* and △*15D*, which participate in fatty acid synthesis, were identified in the fungus (Sakuradani et al., 1999), plants (Vrinten et al., 2005; Lee et al., 2016), and animals except for mammals (Zhou et al., 2011). Therefore, it is necessary to consider whether the *SCD* and other desaturases all have a function in *Y. lipolytica* that is to promote lipid accumulation.

**FIGURE 3:**
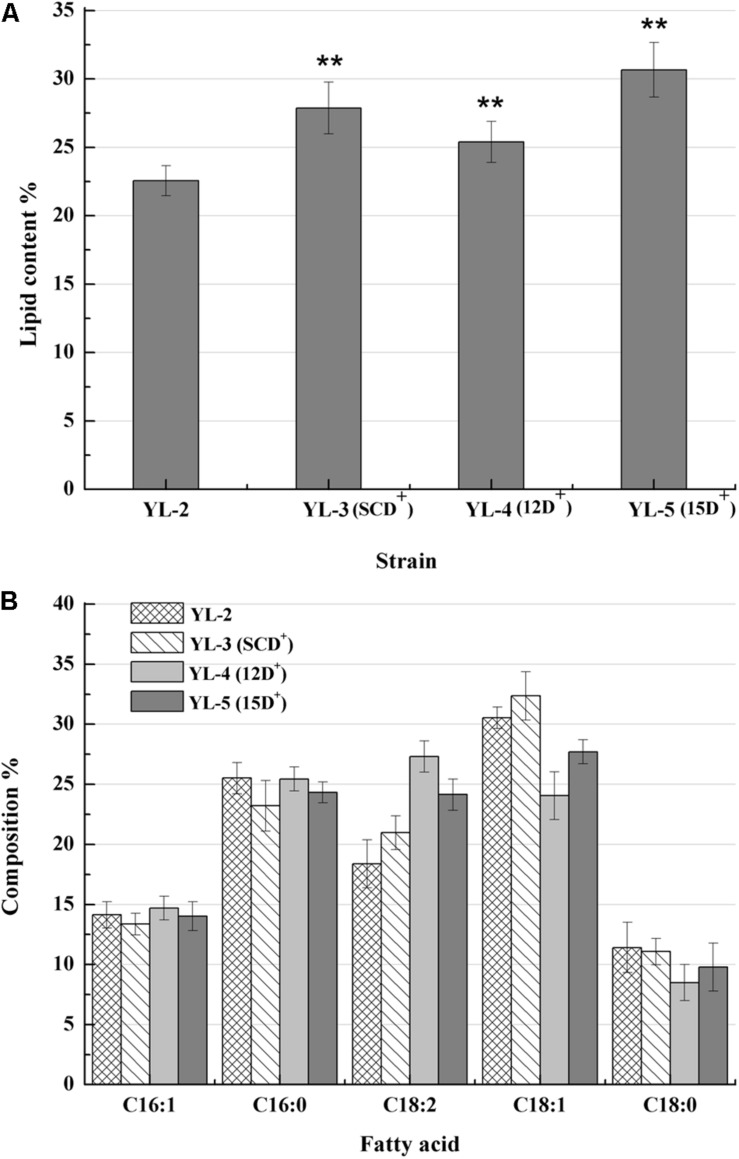
Lipid contents and fatty acid profiles of the strains with the expression of *12D*, *SCD*, and *15D*. **(A)** The percentage of lipid and fatty acid dry cell weight (Lipid% DCW) was shown for strains YL-2, YL-3(SCD^+^), YL-4(12D^+^), and YL-5(15D^+^) cultivated in the media of C:N = 50. **(B)** Fatty acid profiles of lipid extract from the strains YL-2, YL-3(SCD^+^), YL-4(12D^+^), and YL-5(15D^+^) were shown after 6 day shake flask fermentation. Error bars represent standard deviations (*n* = 3). The asterisks indicate a significant difference compared with the control (**p < 0.01).

To evaluate the effect of desaturases on lipid production, plasmids pJN44-*DGA1*-*SCD*, pJN44-*DGA1*-△*12D*, and pJN44-*DGA1*-△*15D* were separately inserted into YL-1, resulting in YL-3(SCD^+^), YL-4(12D^+^), and YL-5(15D^+^), respectively. We found that the lipid production of YL-3(SCD^+^), YL-4(12D^+^), and YL-5(15D^+^) all outperformed the control YL-2, accumulating 27.9, 25.4, and 30.7% lipid content, respectively (Figure 3A). The strain YL-5(15D^+^) was significantly (*P* < 0.01) higher than the other strains. The fatty acid profiles of YL-3(SCD^+^), YL-4(12D^+^), and YL-5(15D^+^) strains were presented in Figure 3B. For YL-3(SCD^+^), the levels of C18:1 and C18:2 increased. For YL-4(12D^+^), the level of C18:2 increased. For YL-5(15D^+^) strain, the C18:2 level increased from 18.5 to 24.2%, but less than that of the YL-4(12D^+^). Overexpression of *SCD*, △*12D*, and expression of △*15D* increased lipid production, and △*15D* achieved higher lipid production. Furthermore, the desaturase increased unsaturated fatty acids content, which is beneficial for biodiesel production.

### Combination of Different Desaturases for Improved Lipogenesis

**FIGURE 4:**
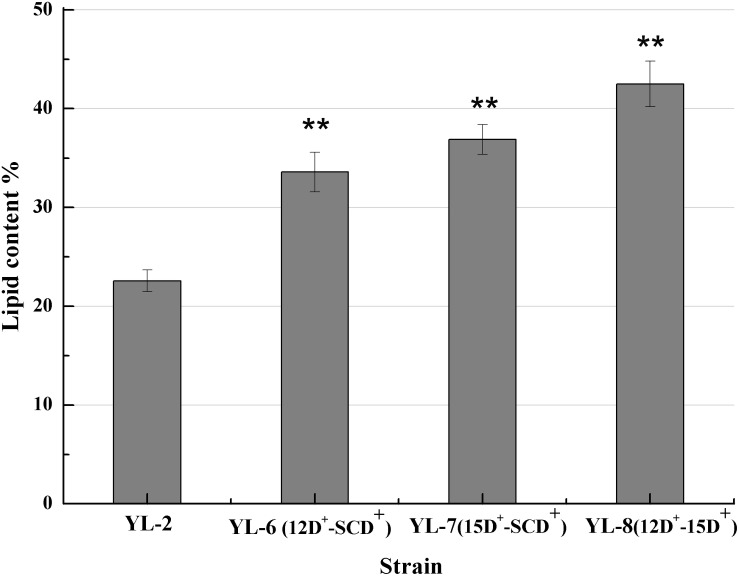
Lipid contents of the strains with the expression of *12D*-*SCD*, *15D*-*SCD*, and *12D*-*15D*. Error bars represent standard deviations (*n* = 3). The asterisks indicate a significant difference compared with the control (**p < 0.01).

The previous experiments showed that desaturases play an important role in lipid accumulation. Therefore we hypothesized that increasing the copy number of the desaturases would further improve lipid synthesis. To assess the effect of co-expression of *SCD* and △*12D*, *SCD* and △*15D*, and △*12D* and △*15D* on lipid production, plasmids pJN44-*DGA1*-*12D*-*SCD*, pJN44-*DGA1*-*15D*-*SCD*, and pJN44-*DGA1*-*12D*-*15D* were inserted into YL-1, resulting in strains YL-6(12D^+^-SCD^+^), YL-7(15D^+^-SCD^+^), and YL-8(12D^+^-15D^+^), respectively. All of these strains outperformed the control, accumulating 33.58%, 36.87%, and 42.57% of lipid, respectively (Figure 4). The lipid production of the engineered strain YL-8 increased the most. We assayed the strains for fatty acid profiles and saw C16:1, C16:0, C18:2, C18:1, and C18:0 fatty acids (Table 2) predominantly. Compared with the YL-2 control, the C18:2 content of the YL-6(12D^+^-SCD^+^) strain increased from 19.0 to 27.3%, but the C18:1 content decreased slightly. This result suggested that the △*12D* had a higher activity to convert C18:1 to C18:2, compared to the *SCD*. The C18:1 content of the YL-7(15D^+^-SCD^+^) strain increased from 27.5 to 32.8%, while simultaneously, the content of C18:2 showed a slight increase, which suggested that the *SCD* has a higher activity to convert C18:0 to C18:1. Compared with the control, the C18:2 content of the YL-8(12D^+^-15D^+^) strain increased by 64.3%, but the content of C18:1 was reduced. These results suggested that Δ*12D* in *Y. lipolytica* has the highest activity, followed by *SCD* and Δ*15D*. As described above, co-expressing two desaturases improved the lipid production over the strains of overexpressing a single desaturase. Moreover, the simultaneous expression of △*15D* and △*12D* exhibited the highest lipid production.

**TABLE 2:** Fatty acid profiles as a% of total fatty acids.

**Strains**	**Fatty acid accumulation (%)**				
	
	**C16:1**	**C16:0**	**C18:2**	**C18:1**	**C18:0**
**YL-2**	15.88 ± 0.9	28.08 ± 2.1	19.04 ± 1.4	27.52 ± 2.0	9.50 ± 1.1
**YL-6(12D^+^-SCD^+^)**	14.61 ± 1.0	25.22 ± 0.8	27.29 ± 1.0	24.21 ± 1.2	8.66 ± 2.0
**YL-7(15D^+^-SCD^+^)**	14.37 ± 0.9	23.54 ± 1.1	19.29 ± 1.2	32.80 ± 0.7	10.01 ± 1.0
**YL-8(12D^+^-15D^+^)**	14.49 ± 1.0	23.38 ± 0.8	31.29 ± 1.5	20.51 ± 1.4	10.33 ± 1.0

### Overexpression of *ACC1* in the Engineered Strain Led to Significant Increase in Lipid Accumulation

**FIGURE 5:**
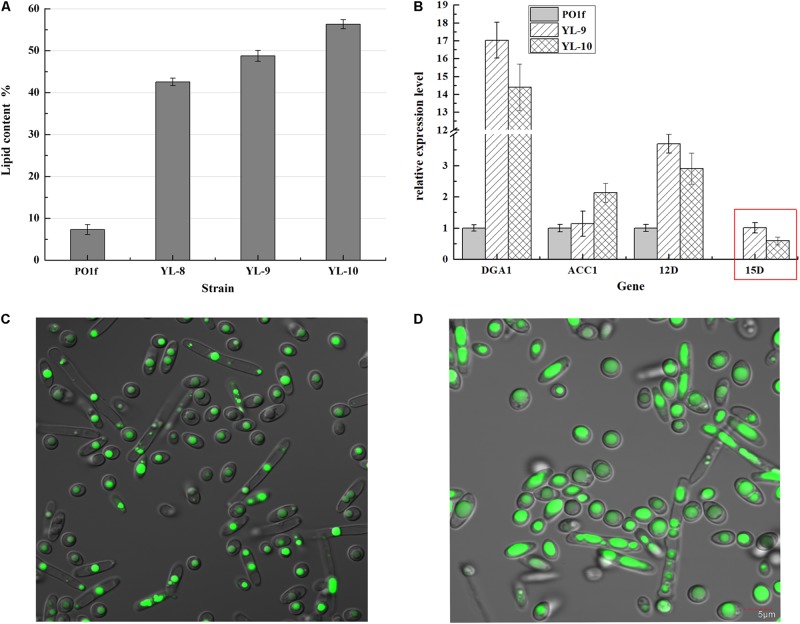
Combinatorial analysis of the PO1f, YL-8(12D^+^-15D^+^), YL-9, and YL-10 strains. **(A)** The percentage of lipid and fatty acid dry cell weight (Lipid% DCW) was shown for strains PO1f, YL-8(12D^+^-15D^+^), YL-9, YL-10 cultivated in the media of C:N = 50. **(B)** Relative quantification of RNA transcripts using real-time PCR. *ACTIN* was used as the reference gene. Error bars represent standard deviations (*n* = 3). The data are normalized by the transcription level of genes in PO1f, except for △15D, which is normalized by the transcriptional level in YL-9. Red box: the transcriptional levels of △*15D* in YL-9 and YL-10 were calculated using the 2^–△ct^ calculation (△ct = C_T_, _Target_ − C_T_, _Actin_). **(C)** Imaging analyses of the PO1f strain at 48 h of the shake flask fermentation period. **(D)** Imaging analyses of the YL-10 strain at 48 h of the shake flask fermentation period.

The above strategies show that all three desaturases were positive effectors of lipogenesis, and overexpressing two copies of desaturases exhibited higher lipid production. However, the *DGA1*, △*12D*, and △*15D* genes in the YL-8(12D^+^-15D^+^) strain were expressed episomally, which rendered YL-8(12D^+^-15D^+^) unstable. Thus, we constructed the YL-9 strain using the integrative cassette (*12D*-*15D*-*DGA1*-*MFE1*-up-loxp-down) to insert those three genes into the chromosome of YL-1 in the *MFE1* site, to obtain the genetically stable strain, YL-9. *MFE1* encodes the multifunctional enzyme involved in the second step of the β-oxidation pathway (Dulermo and Nicaud, 2011). The deletion of gene *MFE1* has been shown to reduce fatty acid degradation and promote lipid accumulation in *Y. lipolytica* (Blazeck et al., 2014). The engineered strain YL-9 accumulated lipid at 48.78% of DCW, compared to 42.57% for the strain YL-8(12D^+^-15D^+^) (Figure 5A).

*DGA1* encodes diacylglycerol acyltransferase type 2, which involved in the final step of TAG synthesis. Overexpression of *DGA1* is known to increase the lipid content in *Y. lipolytica*. For example, overexpression of *DGA1* produced the strain NS297, which exhibited a two-fold increase in lipid content compared to the wild type *Y. lipolytica* (Friedlander et al., 2016). However, if only *DGA1* were modulated, it can lead to an imbalance between fatty acid and TAG synthesis pathway, which adversely affects the cellular fatty acid composition and cell growth. Co-expressing *ACC1* and *DGA1* increased throughput in both fatty acid and TAG synthesis pathways simultaneously without intermediate accumulation (Tai and Stephanopoulos, 2013). Thus, we overexpressed *ACC1* in the strain YL-9 to enhance fatty acid production. Plasmid pJN44-*ACC1* was inserted into YL-9, yielding strain YL-10. The YL-10 was able to achieve 56.34% lipid content, an increase of 15% compared to YL-9, and a 6.8-fold improvement over the control PO1f (Figure 5A). These results demonstrated that deletion of *MFE1* increased lipid accumulation by 14.6%, and overexpression of *ACC1* in the strain YL-9 also improved the lipid production, presumably due to a better balance between the fatty acid and TAG synthesis pathway.

The transcriptional level of these inserted genes was also measured by qRT-PCR of total RNA (Figure 5B). Compared with YL-9, *ACC1* overexpression increased the transcriptional levels of *ACC1* by 87% in YL-10, which might be attributed to improving the lipid content by 15%. However, compared with YL-9, the transcriptional levels of △*12D* and △*15D* genes decreased in YL-10, which did not reduce the lipid content. This result suggested that the *ACC1* gene was the most effective gene for producing lipid. Moreover, overexpression of *ACC1* under TEF promoter in YL-10 is only twice that of the control strain PO1f, whereas overexpression of *DGA1* is 17 times higher. The reason was that the native promoter of *ACC1* is stronger than the native promoter of *DGA1* (Supplementary Figure 3). The transcriptional level of *ACC1* relative to *ACTIN* is 0.6 in PO1f, whereas the transcriptional level of *DGA1* is 0.01 in PO1f.

We used a Nile red-based fluorescence microscopy to determine relative lipid content. The strains PO1f and YL-10 cells were harvested, and the image analyzed during the exponential growth phase (48 h). It is evident that the PO1f cells were exhibiting two phenotypes of spherical and hyphae, and lipid bodies were smaller in the landscape mode (Figure 5C). The vast majority of YL-10 cells retained the spherical phenotype with a larger fat droplet; the lipid reached approximately 2/3rd of cell volume (Figure 5D).

### Fermentation Performance of the Transformant

To further evaluate the cell growth and explore lipid accumulation characteristics of the engineered strain YL-10, the fermentation was conducted using a 5-L stirred-tank bioreactor with the glucose as sole carbon source. Previous studies used the “carbon-rich” mode to conduct their fermentations. This “carbon-rich” mode maintains a relatively high carbon concentration to that of nitrogen such that the excess carbon can lead to lipid synthesis in *Y. lipolytica*. The use of the “carbon-rich” mode resulted in significant carbon loss (Xu et al., 2017). Consequently, we first cultivated the strains with 40 g/L glucose, and then glucose was fed continuously to maintain the low glucose concentration in the reactor, which would help promote cell growth and lipid synthesis. The fermentation experiment was repeated four times, and the results showed that the fermentation was reproducible. We chose one of the fermentation experiments for further analysis.

**FIGURE 6:**
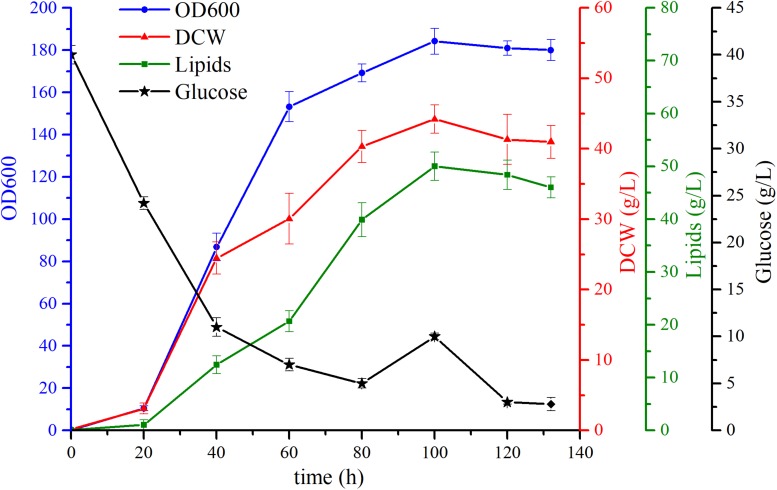
Fermentation characteristics of the YL-10 strains.

For the constructed strain YL-10, 160 g/L of glucose was fully consumed within the 132 h of fermentation, and the final biomass was achieved at 44.2 g/L. Throughout the fermentation, 1.7 g/L/h and 2.5 g/L/h of the glucose consumption rate was observed during the exponential growth phase (20-80 h) and stationary phase (80-120 h), respectively. The lipid production in the YL-10 strain peaked during the stationary phase approaching 50 ± 2.6 g/L. The maximal lipid content was 77.8% of DCW during the stationary phase, a 38% increase in lipid production compared to the shake flask experiment (Figure 6).

## Discussion

Biogenesis of peroxisomes, where β-oxidation takes place, is impaired by *PEX10* deletion (Ledesma-Amaro and Nicaud, 2015). Many studies have demonstrated the promotion of lipid accumulation by knocking out *PEX10*, *MFE1*, and *PEX10* and *MFE1*. Generally, it was clear that the deletion of *PEX10* abolished the peroxisome, resulting in the interruption of the β-oxidation pathway. However, the deletion of both *PEX10* and *MFE1* simultaneously led to a further increase in lipid production. The results suggest that an additional β-oxidation pathway may exist in *Y. lipolytica*. Indeed, a previous study had found that *Y. lipolytica* genome sequence contains an additional NAD^+^ dehydrogenase gene, a potential candidate for vestigial mitochondrial β-oxidation (Haddouche et al., 2011). This result goes against the current view that yeasts have only the peroxisomal β-oxidation capacity (Haddouche et al., 2011). In this study, we lowered fatty-acid catabolism by reducing β-oxidation (*MFE1* deletion) and peroxisomal biogenesis (*PEX10* deletion). Deletion of *PEX10* accumulated lipid at 14.5% of DCW, promoting a 1-fold increase in lipid content. However, the fatty acid composition was also changed, the levels of linoleic acid (C18:2) and oleic acid (C18:1) decreased significantly, which implies that *PEX10* controls the composition of intracellular fatty acid content (Hong et al., 2009). The *MFE1* gene deletion increased the lipid production by 14.6%, from 42.6% to 48.8% of lipid DCW. However, we observed that the *MFE1* deletion had a negative effect on biomass yield.

*ACC1* catalyzes the first committed step toward lipid biosynthesis; it converts cytosolic acetyl-CoA into malonyl-CoA. Malonyl-CoA is the primary precursor for fatty acid elongation. However, *ACC1* overexpression alone in the eukaryotic organisms has been shown to have a minimal improvement in lipid production (Tai and Stephanopoulos, 2013). This result is consistent with the results from the overexpression of the native *ACC1* in *Y. lipolytica*. This result also explains why *ACC1* was not overexpressed independently but overexpressed simultaneously with *DGA1* in the engineered strain. *DGA1* and *DGA2* are the key components of the lipogenic pathway in the final step of TAG synthesis: incorporation of the third acyl-CoA onto the diacylglycerol backbone. *DGA1* or *DGA2* overexpression has been shown to promote lipid accumulation in the plant seeds, *Rhodosporidium toruloides*, and *Y. lipolytica* (Tai and Stephanopoulos, 2013; Friedlander et al., 2016; Zhang et al., 2016; Meyer and Stecca, 2017). Friedlander et al. engineered the strain NS297 through overexpression of endogenous *DGA1* in *Y. lipolytica* and the result indicated the strain NS297 exhibited a two-fold increase in lipid content compared to the wild type as evaluated by fluorescence assay (Friedlander et al., 2016).

Furthermore, the engineered *Y. lipolytica* strains with overexpressed heterologous *DGA1* genes (*R. toruloides DGA1*, *L. starkeyi DGA1*) showed their lipid levels were approximately three-fold higher than the wild type and were also higher than transformants overexpressing the native *Y. lipolytica DGA1* gene (Salunke et al., 2015; Zhang et al., 2016). In this study, we showed that *DGA1* overexpression resulted in the lipid content of 22.56%, exhibiting a two-fold improvement over control (Figure 2A). These results indicated that *DGA1* is the most critical gene involved in lipid production. Only overexpression of the *DGA1* (native or heterologous *DGA1* from *R. toruloides*) could achieve a significant increase in lipid production. Co-expressing *ACC1* and *DGA1* markedly increased lipid production through simultaneously regulate both upstream and downstream pathways without intermediates accumulation to further enhance the lipid accumulation. Another effective method to increase cellular lipid is by removing the β-oxidation capacity, and many studies have achieved elevated fatty acid production by knocking out different genes in the β-oxidation pathway (Dulermo and Nicaud, 2011; Haddouche et al., 2011).

By expressing *SCD*, △*12D*, and △*15D* genes in *Y. lipolytica*, we demonstrated that manipulating the desaturases in the lipid metabolism pathway could promote lipid accumulation. To evaluate the relationships among fatty acid desaturase proteins of *SCD*, △*12D*, and △*15D*, we compared the amino acid sequences of △*12D* and △*15D* to that of *SCD*. The sequence of △*12D* and △*15D* shared 47% and 45% identity with the amino acid sequence of *SCD*, respectively. Thus, overexpression of *SCD*, △*12D*, and expression of △*15D* can improve lipid production. Moreover, the ratio of unsaturated to saturated fatty acid in YL-6(12D^+^-SCD^+^), YL-7(15D^+^-SCD^+^), and YL-8(12D^+^-15D^+^) strains is 1.95, 1.98, and 1.97, respectively (Table 2). Compared to that of YL-2 strain (the ratio is 1.66), the desaturases increased unsaturated fatty acids content, and the fatty acid composition is different. This result demonstrated that the desaturases affect the lipid content and fatty acid composition. Unfortunately, the engineered strain expressing the △*15D* (from flax) did not produce α-linolenic acid (ALA; 18:3 ω3) in *Y. lipolytica*. Wang et al. showed that the expression of *F. moniliforme* ω3 desaturase enabled the production of ALA to more than 28% of total fatty acids in the wild type *Y. lipolytica* (Wang et al., 2013). This result suggests that the non-fungal ω3 desaturase (△*15D* from flax) has a weak activity to convert linoleic acid to ALA. However, the transformant expressing △*15D* exhibited a notable increase in lipid content. Therefore, the *SCD*, △*12D*, and △*15D* desaturases all increased fatty acid synthesis and produced the corresponding product. This study provides a promising strategy for increasing the synthesis of lipids in *Y. lipolytica*, which is beneficial for biodiesel production. This strategy develops the path for microbial oil overproduction to lipid biosynthesis. Moreover, the lipid productivity of 50 g/L and lipid content of 77.8% DCW are obtained by the fermentation, which will lay a theoretical foundation for large-scale production.

## Data Availability Statement

All datasets generated for this study are included in the article/Supplementary Material.

## Author Contributions

FY, GD, and YM designed the study. FY, GD, SQ, and YN carried out the experiment. FY, GD, SQ, YN, CH, and YM analyzed the data and wrote the manuscript.

## Conflict of Interest

SQ and YN were employed by the Xi’an Healthful Biotechnology Co., Ltd. The remaining authors declare that the research was conducted in the absence of any commercial or financial relationships that could be construed as a potential conflict of interest.
