# Supplementary material for: Overexpression of △12, △15-Desaturases for Enhanced Lipids Synthesis in Yarrowia lipolytica
Source: Front Microbiol. 2020 Feb 25;11:289. doi: 10.3389/fmicb.2020.00289 (PMC7051990; doi:10.3389/fmicb.2020.00289)
Supplement: Supplementary file 1 [file Data_Sheet_1.docx]

**Overexpression of △12, △15-desaturases for enhanced lipids synthesis in *Yarrowia lipolytica***

Feng Xin Yan^1^, Gui Ru Dong^2^, Shan Qiang^2,3^, Yong Jie Niu^3^,

Ching Yuan Hu^2,4^ and Yong Hong Meng^2*^

^1^ College of Mechanical and Electronic Eng., Northwest A&F University, Xinong Road, 22, Yangling, Shaanxi 712100, P. R. China

^2^ Shaanxi Engineering Lab for Food Green Processing and Security Control, College of Food Engineering and Nutritional Science, Shaanxi Normal University, 620 West Chang’an Avenue, Chang’an, Xi’an 710119, P. R. China

^3^ Xi’an Healthful Biotechnology Co., Ltd., HangTuo Road, Chang’an, Xi’an 710100, P. R. China

^4^ Department of Human Nutrition, Food and Animal Sciences, College of Tropical Agriculture and Human Resources, University of Hawaii at Manoa, 1955 East-West Road, AgSci. 415J Honolulu, HI 96822, USA

^*^ Corresponding author:

Yong Hong Meng, Tel.: +086 029 85310517, E-mail: [mengyonghong@snnu.edu.cn](mailto:mengyonghong@snnu.edu.cn)

**Supplementary Table 1.** List of genes and enzymes used in this study.

| Genes and enzymes |  |
| --- | --- |
| Genomic backgrounds |  |
| Name | **Genotype (function of knockout)** |
| POlf | MatA, leucine, uracil, no extracellular proteases |
| *PEX10* | POlf-△*PEX10* (prevents peroxisome biogenesis) |
| *PEX10 MFE1* | POlf-△*PEX10*-△*MFE1* (prevents peroxisome biogenesis and β-oxidation) |
| Enzymatic overexpression |  |
| Name | **Function** |
| DGA1 | Convert MG to DG |
| SCD | Convert stearoyl-CoA to oleoyl-CoA |
| △12 desaturase | Convert oleoyl-CoA to linoleoyl-CoA |
| △15 desaturase | Convert linoleic acid to α-linolenic acid (ALA) |
| ACC1 | Convert Acetyl-coA carboxylase (ACC1) to malonyl-CoA |
| Auxotrophic makers |  |
| Name | **Utilized for expression** |
| Leucine^+/-^ | Episomally or chromosomally |
| Uracil^+/-^ | chromosomally |

**Supplementary Table 2.** List of primers used in this study.

| primers | sequence |
| --- | --- |
| *PEX10*-up1 | GTCAT**GGGCCC**TAGTAAGTGGTGCTCTGG |
| *PEX10*-up2 | GATCG**TCTAGA**ACGAAGATTCCGAGGGGAT |
| *PEX10*-down1 | TCGAC**ACTAGT**TAGCATTGGAGATATGAGTGG |
| *PEX10*-down2 | AGTCA**CATATG**AAGAAGTTCTCACACAGCA |
| *PEX10*-test1 | AGACAGATTTTGACCAGTAATC |
| *PEX10*-test2 | TCATTGCATGTAGCCAGTGG |
| Ura3-testF | TCCTGGAGGCAGAAGAACTT |
| Ura3-testR | ATACGTGAGTCAGAAGGGCT |
| *MFE1*-up1 | ATCGA**GGGCCC**TACAAACTGTGTGCTTGTC |
| *MFE1*-up2 | TAGAT**CCGCGG**GATTTAGACTCTGCCTCGG |
| *MFE1*-down1 | CTACG**ACTAGT**AAGTGATACGAGTTAAGTAAATT |
| *MFE1*-down2 | TCAGA**CATATG**GATACTGTTTGTCCATTGAT |
| *MFE1*-test1 | ATGCAACAAGCCGAGAGAATC |
| *MFE1*-test2 | GCCATGTCTCCCACCTTTTC |
| *DGA1*-for. | ACTGA**AAGCTT**ATGACTATCGACTCACAATACT |
| *DGA1*-rev. | CTGAT**CCCGGG**TTACTCAATCATTCGGAACT |
| *SCD*-for. | GTACT**AAGCTT**ATGGTGAAAAACGTGGACCAAGTGG |
| *SCD*-rev. | AGTCA**CTGCAG**CTAAGCAGCCATGCCAGACAT |
| *12D*-for. | GCTCAA**AGCTTA**TGGATTCGACCACGCAGA |
| *12D*-rev. | CTGAC**CCCGGG**CTACTTTTTAGAAGGCAGGCC |
| *ACC1*-for. | TGCTGCAGGTCGACTCTCCCATGCGACTGCAATTGAGGACACTAACAC |
| *ACC1*-rev. | GGCAAACTATCTGTTAACCCTCACAACCCCTTGAGCAGCTCAGCCCGCTCAG |
| *Actin*-RT-for. | TCCAGGCCGTCCTCTCCC |
| *Actin*-RT-Rev. | GGCCAGCCATATCGAGTCGCA |
| *DGA1*-RT-for. | AACGGAGGAGTGGTCAAGCGA |
| *DGA1*-RT-Rev. | TTATGGGGAAGTAGCGGCCAA |
| *ACC1*-RT-for. | GCTTCTCACGGAGGTCATACAGT |
| *ACC1*-RT-Rev. | CTGCGGCAATACCGTTGTTAG |
| *SCD*-RT-for. | AACGTGGACCAAGTGGATCT |
| *SCD*-RT-rev. | CCGGAGGTGTACTTGACCTT |
| *12D*-RT-For. | CATGACTGGTCTGAGCTTGC |
| *12D*-RT-rev. | GTGGTTGACAGCCCATTTGT |
| *15D*-RT-for. | GTGCTAAGAAGCTGCTGGAC |
| *15D*-RT-rev. | GGAATAGCGGCTCGAATGTC |

Sequence in bold and underline indicates restriction enzyme sites (see text for details).

**Supplementary Figure 1.** The maps for plasmids pJN43 (P_TEF_-Txpr2) and pJN44 (P_TEF_-Txpr2) used in this study.

**
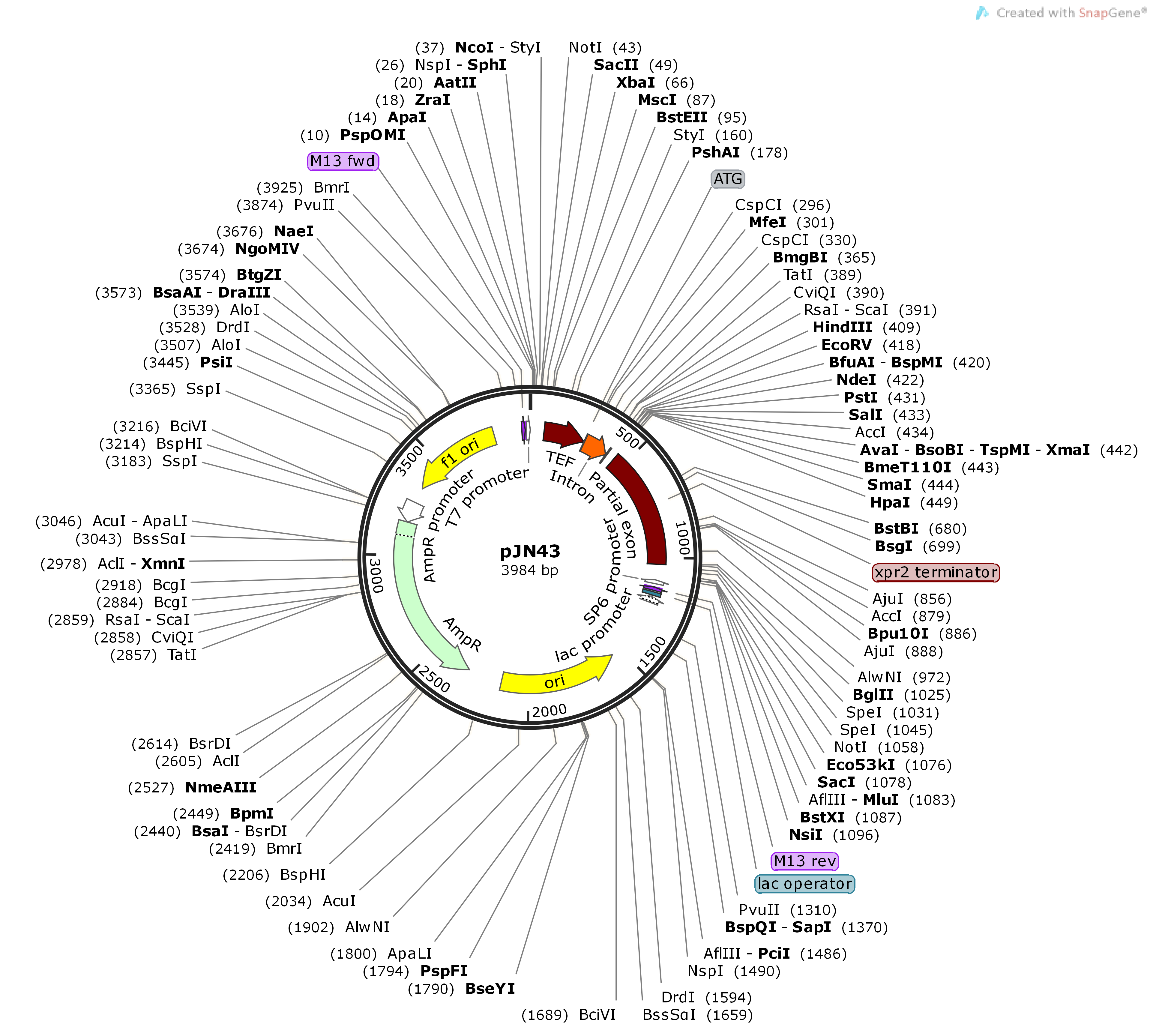
**


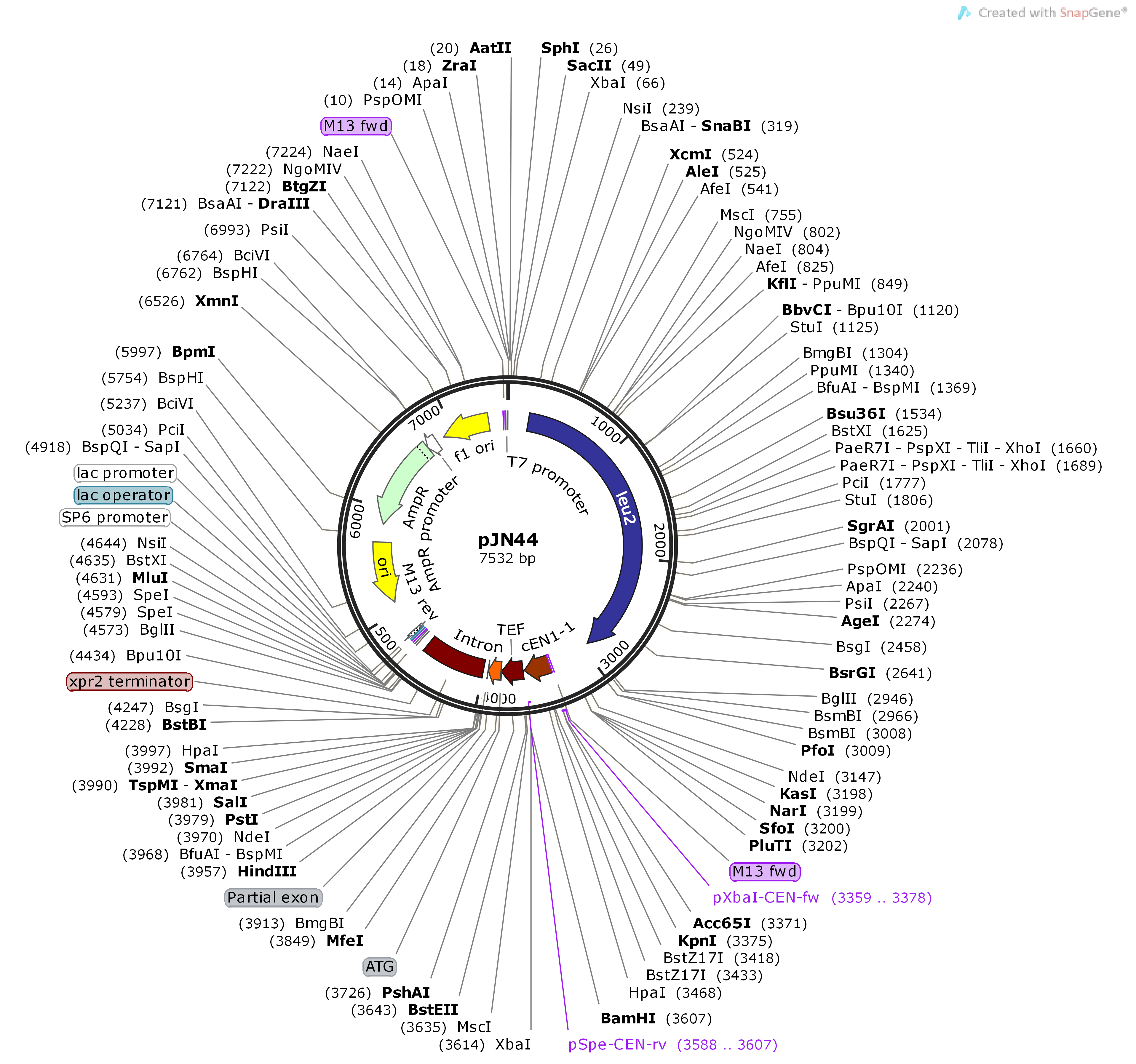


**Supplementary Figure 2.** The map for plasmid pLoxp-ura-loxp used in this study.


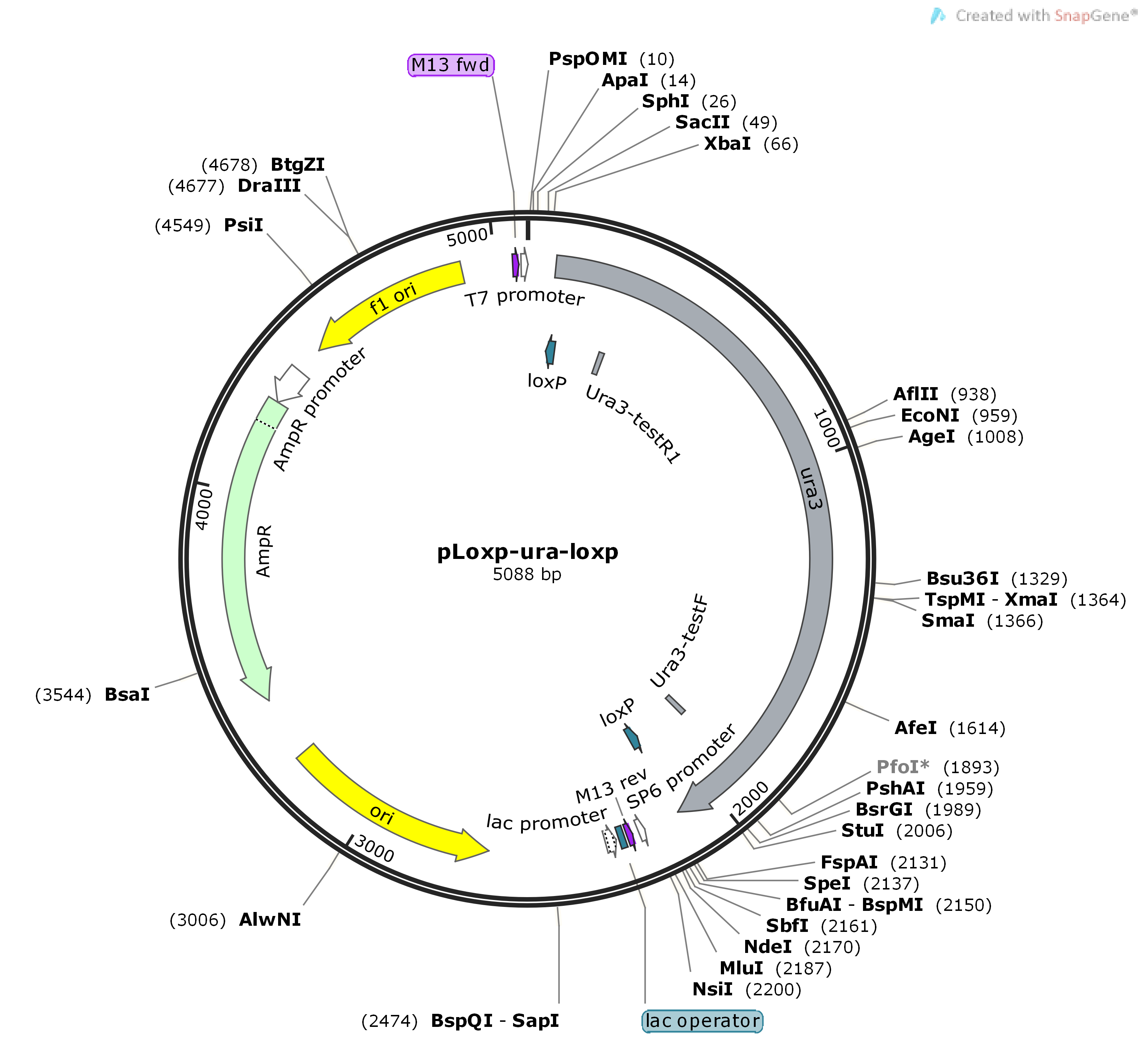


**Supplementary Figure 3.** Relative quantification of RNA transcripts using RT-PCR. The transcriptional levels of genes were calculated using the 2^-△ct^ calculation (△ct = C_T_, _Target_ - C_T_, _Actin_) in PO1f, YL-9 and YL-10.
